# Supplementary material for: Multi‐dataset identification of innovative feature genes and molecular mechanisms in keratoconus
Source: J Cell Mol Med. 2024 Sep 19;28(18):e70079. doi: 10.1111/jcmm.70079 (PMC11412914; doi:10.1111/jcmm.70079)
Supplement: Supplementary file 2 — Material S2. [file JCMM-28-e70079-s002.zip › 7.GSEA analysis/1.ARL11/KEGG_PEROXISOME.html]

Details for gene set KEGG\_PEROXISOME[GSEA]

|  || Dataset | ARL11.group.cls#h\_versus\_l |
| Phenotype | group.cls#h\_versus\_l |
| Upregulated in class | h |
| GeneSet | KEGG\_PEROXISOME |
| Enrichment Score (ES) | 0.2423542 |
| Normalized Enrichment Score (NES) | 0.9466289 |
| Nominal p-value | 0.55130786 |
| FDR q-value | 1.0 |
| FWER p-Value | 1.0 |
Table: GSEA Results Summary

  

Fig 1: Enrichment plot: KEGG\_PEROXISOME      
 Profile of the Running ES Score & Positions of GeneSet Members on the Rank Ordered List

  

| SYMBOL | TITLE | RANK IN GENE LIST | RANK METRIC SCORE | RUNNING ES | CORE ENRICHMENT || 1 | BAAT | na | 614 | 0.452 | 0.0595 | Yes |
| 2 | HAO1 | na | 1788 | 0.312 | 0.0789 | Yes |
| 3 | SLC27A2 | na | 2836 | 0.248 | 0.0911 | Yes |
| 4 | ABCD4 | na | 3044 | 0.239 | 0.1259 | Yes |
| 5 | FAR2 | na | 3513 | 0.219 | 0.1497 | Yes |
| 6 | AMACR | na | 4496 | 0.183 | 0.1526 | Yes |
| 7 | NUDT12 | na | 5321 | 0.157 | 0.1556 | Yes |
| 8 | FAR1 | na | 5489 | 0.152 | 0.1767 | Yes |
| 9 | PEX11A | na | 6026 | 0.137 | 0.1846 | Yes |
| 10 | PEX12 | na | 7293 | 0.106 | 0.1660 | Yes |
| 11 | HAO2 | na | 7381 | 0.104 | 0.1813 | Yes |
| 12 | CRAT | na | 7508 | 0.102 | 0.1950 | Yes |
| 13 | NUDT19 | na | 7746 | 0.097 | 0.2048 | Yes |
| 14 | DDO | na | 7947 | 0.093 | 0.2150 | Yes |
| 15 | PXMP4 | na | 8770 | 0.078 | 0.2046 | Yes |
| 16 | MVK | na | 8857 | 0.077 | 0.2153 | Yes |
| 17 | PEX11G | na | 8923 | 0.076 | 0.2264 | Yes |
| 18 | HMGCL | na | 9012 | 0.075 | 0.2366 | Yes |
| 19 | ACOX1 | na | 9236 | 0.071 | 0.2424 | Yes |
| 20 | PEX5 | na | 10066 | 0.059 | 0.2286 | No |
| 21 | XDH | na | 10277 | 0.057 | 0.2322 | No |
| 22 | PEX1 | na | 10678 | 0.051 | 0.2294 | No |
| 23 | PEX26 | na | 10882 | 0.049 | 0.2319 | No |
| 24 | CROT | na | 10944 | 0.048 | 0.2383 | No |
| 25 | PEX11B | na | 12556 | 0.031 | 0.1971 | No |
| 26 | ACSL1 | na | 12605 | 0.030 | 0.2009 | No |
| 27 | PMVK | na | 12782 | 0.029 | 0.2008 | No |
| 28 | EPHX2 | na | 14165 | 0.017 | 0.1639 | No |
| 29 | GNPAT | na | 14371 | 0.016 | 0.1607 | No |
| 30 | PAOX | na | 14391 | 0.016 | 0.1629 | No |
| 31 | ACSL5 | na | 14433 | 0.016 | 0.1644 | No |
| 32 | PEX16 | na | 14820 | 0.012 | 0.1554 | No |
| 33 | ACSL4 | na | 15066 | 0.011 | 0.1501 | No |
| 34 | AGPS | na | 15082 | 0.011 | 0.1515 | No |
| 35 | MPV17 | na | 15145 | 0.010 | 0.1514 | No |
| 36 | CAT | na | 15314 | 0.009 | 0.1481 | No |
| 37 | ACAA1 | na | 15725 | 0.006 | 0.1372 | No |
| 38 | ECH1 | na | 16074 | 0.003 | 0.1278 | No |
| 39 | HACL1 | na | 16289 | 0.002 | 0.1219 | No |
| 40 | MLYCD | na | 16660 | -0.001 | 0.1114 | No |
| 41 | ABCD3 | na | 17290 | -0.005 | 0.0941 | No |
| 42 | PRDX1 | na | 17382 | -0.006 | 0.0925 | No |
| 43 | PEX2 | na | 17424 | -0.006 | 0.0924 | No |
| 44 | PRDX5 | na | 17470 | -0.006 | 0.0922 | No |
| 45 | SCP2 | na | 17655 | -0.008 | 0.0883 | No |
| 46 | PEX14 | na | 17716 | -0.008 | 0.0879 | No |
| 47 | GSTK1 | na | 18167 | -0.012 | 0.0769 | No |
| 48 | ACOX3 | na | 18292 | -0.012 | 0.0754 | No |
| 49 | PEX10 | na | 18753 | -0.015 | 0.0648 | No |
| 50 | PHYH | na | 18808 | -0.016 | 0.0659 | No |
| 51 | SOD1 | na | 19015 | -0.017 | 0.0628 | No |
| 52 | HSD17B4 | na | 19202 | -0.018 | 0.0606 | No |
| 53 | SLC25A17 | na | 19663 | -0.022 | 0.0510 | No |
| 54 | DECR2 | na | 20037 | -0.024 | 0.0443 | No |
| 55 | PEX19 | na | 20183 | -0.025 | 0.0444 | No |
| 56 | PEX7 | na | 21079 | -0.032 | 0.0240 | No |
| 57 | DHRS4 | na | 21842 | -0.038 | 0.0085 | No |
| 58 | PEX3 | na | 21852 | -0.038 | 0.0147 | No |
| 59 | PEX13 | na | 21856 | -0.038 | 0.0211 | No |
| 60 | PECR | na | 23312 | -0.054 | -0.0117 | No |
| 61 | SOD2 | na | 23532 | -0.056 | -0.0084 | No |
| 62 | ACOT8 | na | 23600 | -0.057 | -0.0006 | No |
| 63 | ECI2 | na | 23618 | -0.058 | 0.0087 | No |
| 64 | AGXT | na | 24023 | -0.063 | 0.0078 | No |
| 65 | ACSL3 | na | 24299 | -0.066 | 0.0111 | No |
| 66 | IDH1 | na | 24481 | -0.069 | 0.0177 | No |
| 67 | NOS2 | na | 24516 | -0.070 | 0.0286 | No |
| 68 | EHHADH | na | 24876 | -0.074 | 0.0310 | No |
| 69 | IDH2 | na | 25024 | -0.076 | 0.0398 | No |
| 70 | ABCD1 | na | 25594 | -0.084 | 0.0377 | No |
| 71 | PIPOX | na | 25663 | -0.085 | 0.0503 | No |
| 72 | PEX6 | na | 25912 | -0.089 | 0.0583 | No |
| 73 | ACSL6 | na | 28020 | -0.131 | 0.0200 | No |
| 74 | MPV17L | na | 28727 | -0.150 | 0.0252 | No |
| 75 | ACOX2 | na | 28878 | -0.154 | 0.0472 | No |
| 76 | ABCD2 | na | 29308 | -0.166 | 0.0631 | No |
| 77 | PXMP2 | na | 30188 | -0.194 | 0.0709 | No |
| 78 | DAO | na | 33410 | -0.352 | 0.0380 | No |
Table: GSEA details [plain text format]

  

Fig 2: KEGG\_PEROXISOME      
 Blue-Pink O' Gram in the Space of the Analyzed GeneSet

  

Fig 3: KEGG\_PEROXISOME: Random ES distribution      
 Gene set null distribution of ES for **KEGG\_PEROXISOME**
